# Resilience in Syrian refugee youth

**DOI:** 10.1093/eurpub/ckac129.607

**Published:** 2022-10-25

**Authors:** C Dangmann, Ø Solberg, AK Myhrene Steffenak, S Høye, PN Andersen

**Affiliations:** 1 Faculty of Health and Social Sciences, Inland Norway University of Applied Sciences, Elverum, Norway; 2 Department of Health Science, Swedish Red Cross University, Stockholm, Sweden; 3 Department of Psychology, Inland Norway University of Applied Sciences, Elverum, Norway

## Abstract

**Background:**

The importance of resilience factors in the positive adaptation of refugee youth is widely recognised. However, their actual mechanism of impact remains under-researched. The aim of this study was therefore to explore protective and promotive resilience mechanisms on both negative and positive mental health outcomes. Promotive resilience is seen as a direct main effect and protective resilience as a moderating effect.

**Methods:**

Cross-sectional study with 160 Syrian youth aged 13-24 years, who recently resettled in Norway. A multi-dimensional measure for resilience was used to explore the potential impact of resilience factors on pathways between potentially traumatic events from war and flight (PTE), post-migration stress, mental distress and health-related quality of life (HRQoL). Analyses included regression, moderation and moderated mediation using the PROCESS macro for SPSS.

**Results:**

A direct main effect of resilience factors (promotive resilience mechanism) was found for HRQoL and general mental distress, but not for post-traumatic stress disorder (PTSD). No moderating effects of resilience factors (protective resilience mechanism) were found. Post-migration stressors mediated the effects of PTE, and this indirect effect was present at all levels of resilience. Relational and environmental level resilience factors and combined amounts had more impact than individual level factors.

**Conclusions:**

Despite high risk exposure and mental distress, resilience was also high. The direct main effect of resilience factors and less impact on PTSD, suggests universal resilience building interventions may be beneficial, compared to exclusively targeting groups with high symptom levels. These interventions should target relational and environmental resilience factors as well as individual coping techniques. Additionally, reducing current stress and symptoms could increase the efficacy of resilience factors already present.

**Key messages:**

• Refugee youth may have both high levels of risk and high resilience.

• Universal resilience interventions should focus on relational and environmental support, as well as individual resilience.

